# Enhancing Esthetics in a Complete Denture Patient: Optimizing Results With Different Impression Techniques

**DOI:** 10.7759/cureus.27565

**Published:** 2022-08-01

**Authors:** Akansha V Bansod, Sweta G Pisulkar, Chinmayee Dahihandekar

**Affiliations:** 1 Department of Prosthodontics and Crown & Bridge, Sharad Pawar Dental College and Hospital, Datta Meghe Institute of Medical Sciences, Wardha, IND; 2 Department of Prosthodontics and Crown & Bridge, Sharad Pawar Dental College and Hospital, Wardha, IND

**Keywords:** residual ridge resorption, flabby tissue, cheek plumper, impression techniques, complete denture

## Abstract

Tooth loss followed by complete denture rehabilitation can have significant psychological and social consequences for patients. Dentures restore a sense of normalcy and allow the sufferer to communicate with others in today's image-conscious world. Chewing discomfort, as well as unfavorable aesthetics and phonetics, are the most common denture complaints. A complete denture patient's prosthetic rehabilitation should never be confined to the replacement of lost teeth; rather, the ultimate goal should be the restoration of oral functions and aesthetics. The article describes a straightforward, cost-effective, practical, and aesthetic strategy for rehabilitating a complete denture patient with resorbed ridge, flabby tissue, and sunken cheeks. Thus, an effort has been made to restore the patient’s stomatognathic system. Tooth loss followed by complete denture rehabilitation can have significant psychological and social consequences for patients. Dentures restore a sense of normalcy and allow the sufferer to communicate with others in today's image-conscious world. Chewing discomfort, as well as unfavorable aesthetics and phonetics, are the most common denture complaints. A complete denture patient's prosthetic rehabilitation should never be confined to the replacement of lost teeth; rather, the ultimate goal should be the restoration of oral functions and aesthetics. The article describes a straightforward, cost-effective, practical, and aesthetic strategy for rehabilitating a complete denture patient with resorbed ridge, flabby tissue, and sunken cheeks. Thus, an effort has been made to restore the patient’s stomatognathic system.

## Introduction

Edentulism is a long-term handicap that makes it difficult for edentulous people to do basic functions like eating, speaking, and socializing [1]. As a result of these deficits, some individuals may experience increasing social and psychological problems. Tooth loss also has physical implications, such as atrophy of the supporting alveolar tissues, loss of facial muscle support, and decreased biting force and masticatory efficiency [2,3].

Atwood established that residual ridge resorption is a continual process that occurs after teeth are extracted, resulting in poor jaw architecture and insufficient denture support. Ridge resorption is more common in mandibular ridges and thus major problems with lower dentures are instability, pain, and difficulty in mastication. The chewing ability of the edentulous patient declines by one-fourth of the original masticatory efficiency of the dentulous patient [4,5].

The process of resorption and bone atrophy is slow, occurring over time and not always concurrently between the mucosa and the bone, with the patient adapting to the dentures' instability, but with implications for the dentofacial system's structure and functionality [6]. Traumatic occlusal pressures are known to hasten the elimination of residual ridges. According to Kelly, massive alveolar bone resorption in the maxillary and mandibular arches can result in a movable band of tissue on the crest of the ridge, known as the "flabby ridge," which can cause pain and unretentive dentures. If the flabby ridge is not appropriately handled therapeutically, the true issues come when constructing new dentures. The specific impression procedures of the prosthetic field constitute a viable answer for the success of the mobility treatment if the flabby ridge is kept while fabricating new dentures [7,8].

Dentures restore a natural appearance in our image-conscious world, resulting in enhanced patient confidence and ease in social interactions. Esthetics in Complete denture is not only the exact selection of teeth but the lost facial anatomy. Facial esthetics thus play a critical role [9]. Cheeks are usually the most visible region of the face, supported by teeth, ridges, and muscles, and so play an important role in facial aesthetics. Concavities and hollowing of the cheeks are caused by the loss of molars, total edentulism, an age-related decrease in muscular tone, and weight loss [10]. This causes a loss of or reduction in cheek fullness, giving the face a slumped and emaciated aspect and making the patient appear older, which has negative socio-psychological consequences [11]. In the geriatric population, even after restoring the vertical jaw relation, there is a need for additional support of the oral musculature, which can be augmented by the use of a cheek plumper [12].

This clinical report explains how to treat sunken cheeks, flabby tissue in the maxillary ridge, and a significantly resorbed mandibular ridge with a simpler approach. Detachable cheek plumper can be simply made and added to a conventional maxillary complete denture for functional and aesthetic rehabilitation.

## Case presentation

**Figure 1:**
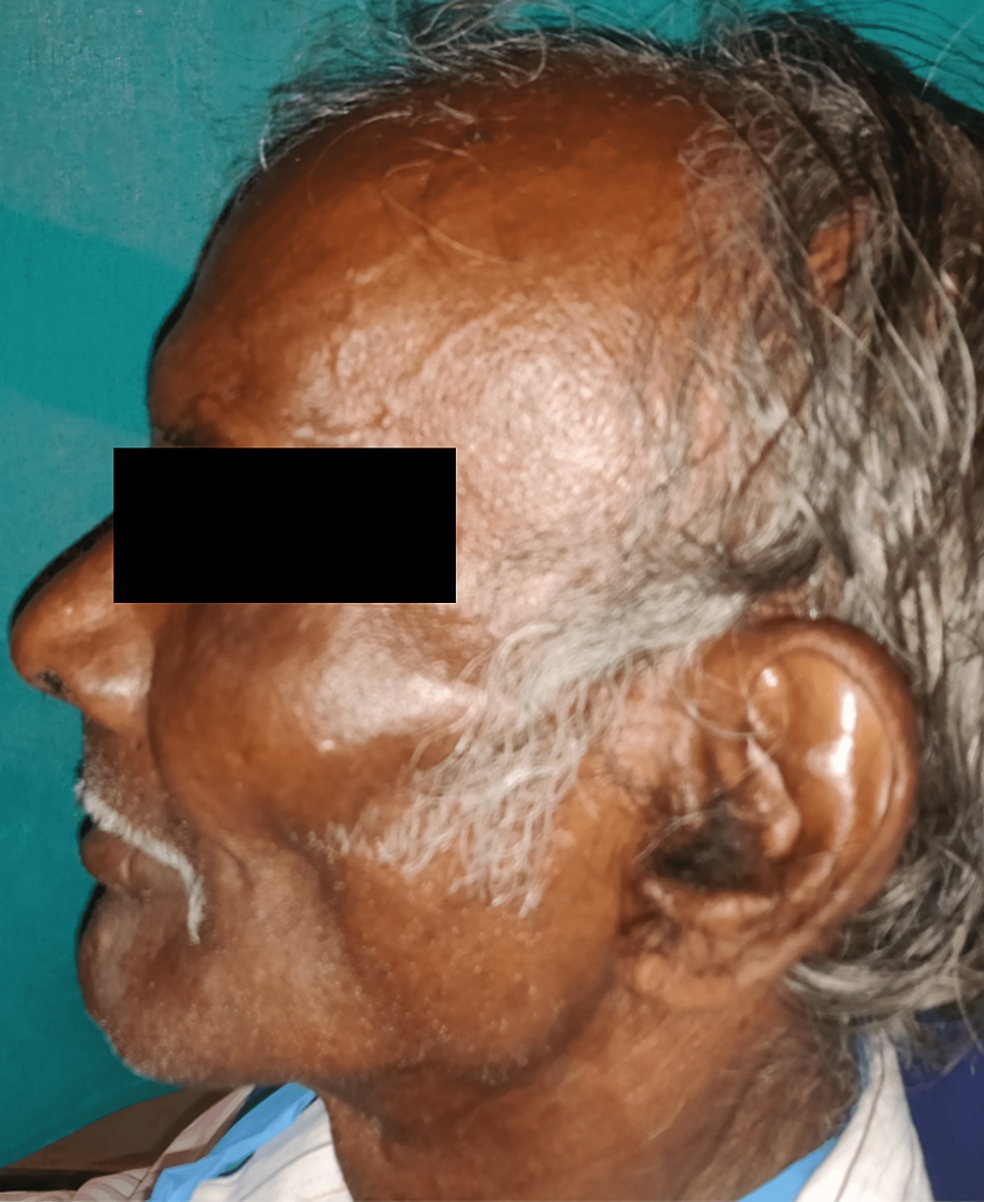
Profile photograph showing sunken cheek appearance

**Figure 2:**
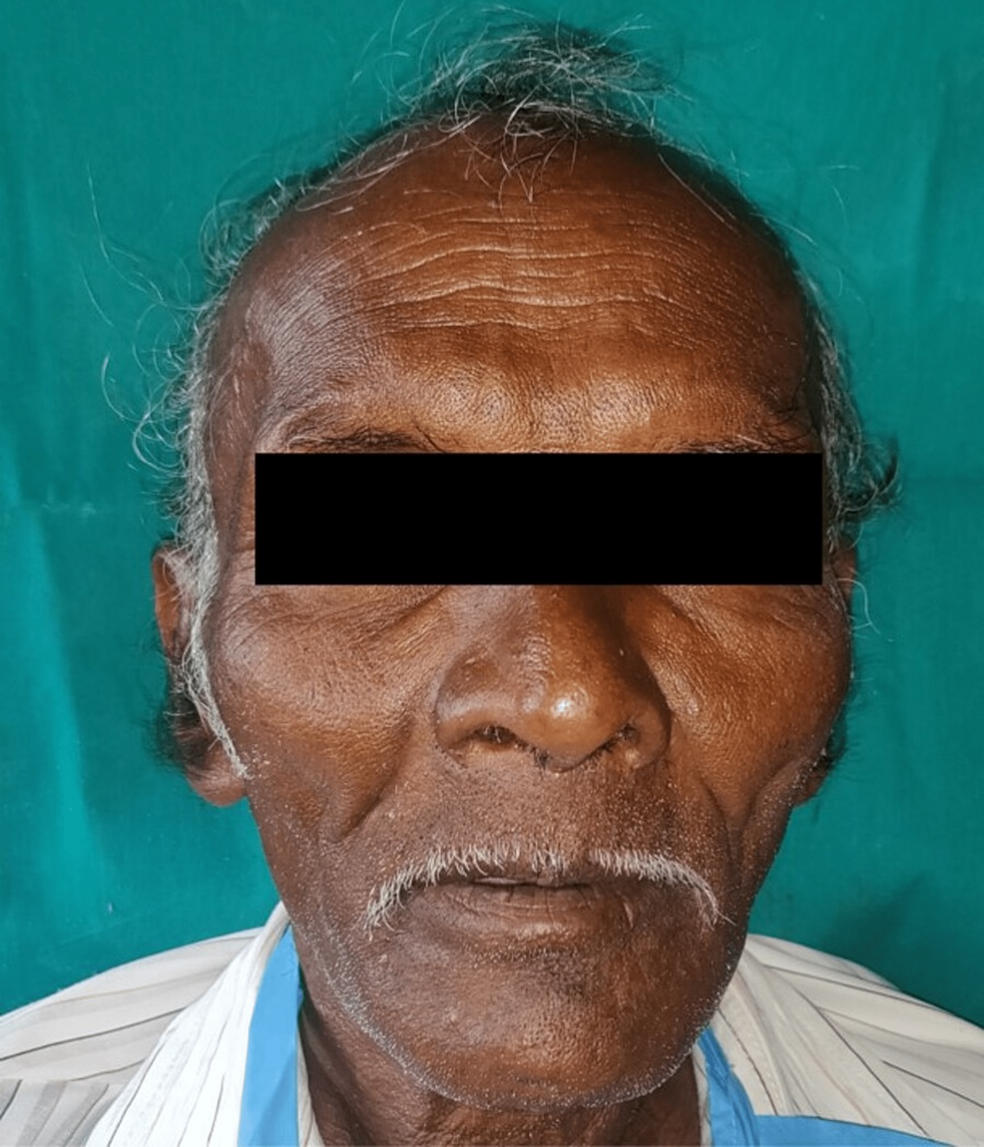
Pre-operative photograph

A 75-year-old male patient reported to the Department of Prosthodontics at Sharad Pawar Dental College and Hospital, Sawangi, Wardha, wanting replacement of missing teeth. His dental history revealed that he had lost his teeth over a period of 2-3 years due to periodontal issues. The patient's main worry was regaining his lost masticatory function, as well as recovering his face fullness that had been lost due to sunken cheeks as seen in the frontal view in Figure 1 and profile view in Figure 2, respectively. 

**Figure 3:**
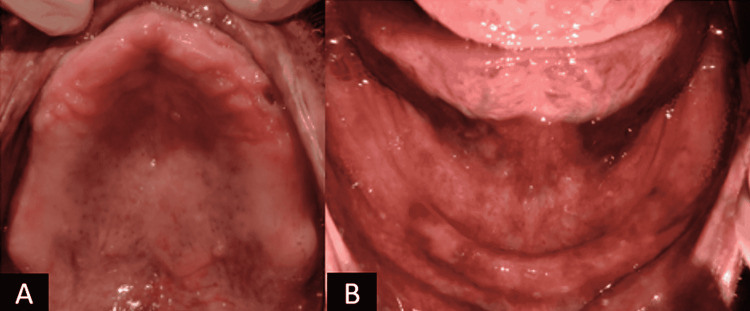
Maxillary and mandibular edentulous arches A: Maxillary edentulous arch; B: Mandibular edentulous arch.

Intra-oral examination of the patient revealed completely edentulous maxillary and mandibular arches (Figure 3).

The flabby tissue was present over the maxillary anterior ridge and severe ridge resorption was seen in the mandibular ridge. The additional oral examination revealed flaccid and unsupported oro-facial musculature, which resulted in drooping and sunken cheeks. The treatment plan was created in response to the patient's requirements.

A complete denture with detachable cheek plumper was developed to solve the issue of sunken cheeks. The impression techniques for resorbed and flabby ridges were modified, and the neutral zone was recorded, followed by teeth setting in the neutral zone. The admix material is manipulated in the patient’s mouth at around 40° C on the lower rim. The patient was instructed to perform routine mandibular movements (including swallowing, sucking of the lips, and pronouncing the vowels), which aided in molding the neutral zone space The steps in the fabrication of complete dentures were as follows:

Step 1. Preliminary Impressions - The maxillary preliminary impression was recorded using the irreversible hydrocolloid impression material (Zhermack Dust-free Thixotropic Tropicalgin, Zhermack SpA, Badia Polesine [RO], Italy) and the mandibular impressions were recorded with admix technique (DPI - Pinnacle Impression Compound and Tracing Sticks, The Bombay Burmah Trading Corporation, Ltd., Mumbai, India).

**Figure 4:**
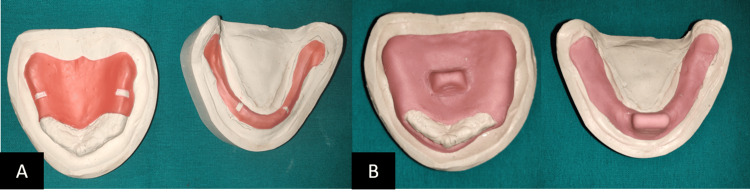
Spacer design and custom tray A: Spacer design; B: Custom tray.

**Figure 5:**
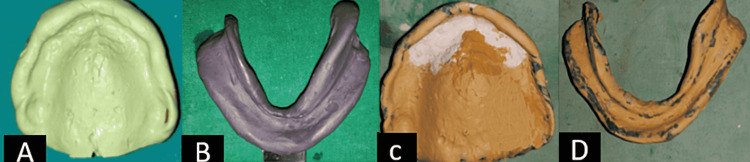
Maxillary and mandibular preliminary impressions and final impressions A: Maxillary preliminary impressions; B: Mandibular preliminary impressions; C: Maxillary final impressions; D: Mandibular final impressions.

Step 2. Final Impressions -, Custom trays were fabricated on the casts obtained from primary impressions (Figure 4). Primary impressions made are shown in Figure 5. They are poured to obtain the primary casts over which custom trays are fabricated, 2 mm short of the sulcus. Border molding with a low fusing impression compound was done and the final impression was made using zinc-oxide impression paste (Figure 5).

A window was created in the area of flabby tissue and painted with the impression plaster, to obtain the flabby tissue in a static state, while the rest of the impression was recorded using zinc oxide eugenol impression paste. Final impressions were inspected for accuracy and poured in vacuum-mixed, type III dental stone to create the master casts. The master casts were poured into dental stone (Zhermack Elite Model Stone, Zhermack SpA, Badia Polesine [RO], Italy) 

**Figure 6:**
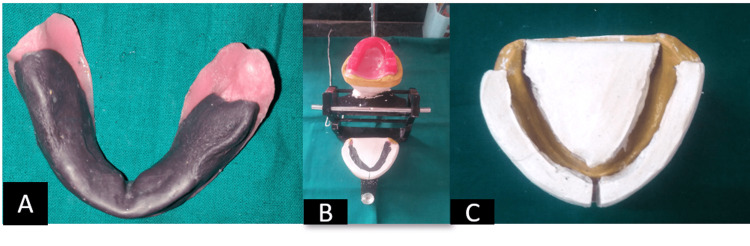
Neutral zone records A: Neutral zone recorded using Admix; B: Jaw relation mounted on three-point articulator.; C: Plaster index records over the neutral zone.

Step 3. Jaw relation records - On a master cast, the auto-polymerizing acrylic resin, cold cure acrylic material (Bombay Burmah Trading Corporation, Ltd., Mumbai, India) was used to create temporary denture bases. Jaw relation was constructed using a preliminary method and articulated utilizing wax occlusal rims. New denture bases were produced with acrylic stops in the molar area that engaged the orthodontic stainless-steel wire to it for retention of the molding material while recording the neutral zone after establishing preliminary vertical dimension and centric relation record (Figure 6).

Following that, impression compound with green stick border molding compound in the ratio of 3:7 was mixed and adapted over the modified mandibular denture base at a predetermined vertical dimension and molded according to muscle action in the neutral zone by asking the patient to do actions such as swallowing and saying words like "oo," "eee."

The impression material was then removed from the base plate, the neutral zone space was retained using plaster index, and molten modeling wax was poured into the gap between the index, taking the shape of the molded occlusal rim in the neutral zone. 

The teeth setting was done on this molded occlusal rim.

**Figure 7:**
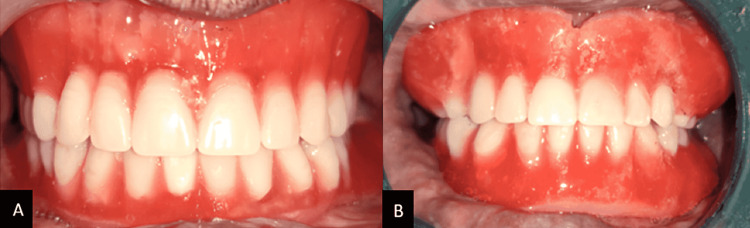
Wax try-in and try-in cheek plumper made of modelling wax attached to maxillary denture A: Wax try-in; B: Try-in cheek plumper made of modelling wax attached to maxillary denture.

Step 4: Try in step - Anatomic teeth were used to arrange artificial teeth. Keeping in mind the chief complaint of the patient, i.e., inability to eat, anatomic teeth were chosen, Various authors have different schools of thought regarding the use of anatomic teeth; there are studies in the literature that have advocated the use of anatomic teeth arranged in bilateral balanced occlusion for improved masticatory efficiency with long term follow-ups and minimal ridge resorption. The mandibular teeth were arranged following the index, and the maxillary teeth were arranged following the mandibular teeth arrangement. In order to preserve the contours established by the plaster indices in the neutral zone, no additional wax was added to the denture flanges. Teeth were arranged in balanced occlusion. Dentures were waxed and aesthetics were used for the try-in (Figure 7).

**Figure 8:**
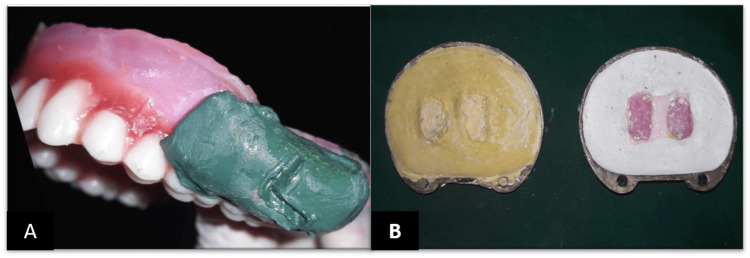
Try-in of cheek plumper A: Green stick was used to mold the material and determine the fullness.; B: Fabrication of cheek plumpers done seperately.

The phonetics, retention, and stability were all examined, but the patient was dissatisfied with the way he looked. Accordingly, at the buccal flange region of the maxillary denture, a bulb of softened green stick wax was added to plump up the sagging cheekbones which were then duplicated into wax once the size of the cheek plumper was determined (Figure 8).

**Figure 9:**
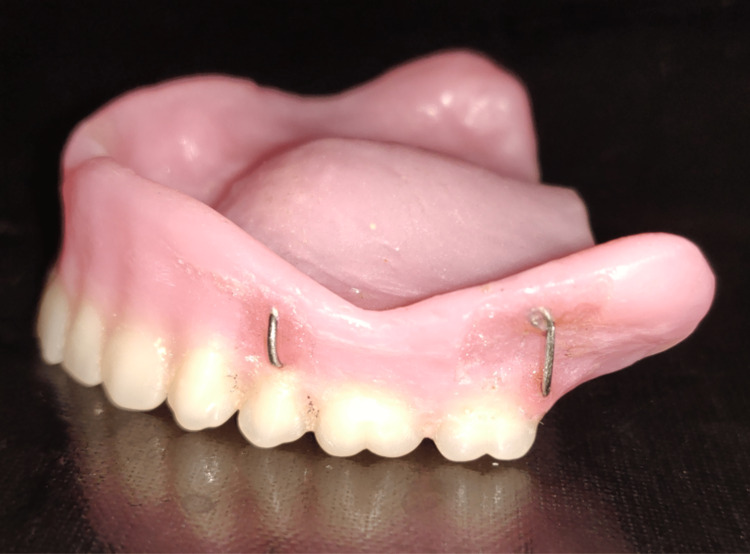
Loop in complete denture

The bulbs added on both sides in the buccal flange region were detached and processed separately along with the complete dentures by compression molding techniques. They were retrieved, finished, and polished. Hooks and loops were attached with the help of auto polymerizing resin. The required cheek support was attained by repeating the operation until the patient was satisfied that the cheeks were adequately supported. Two hooks were inserted within the wax cheek plumper so that they could latch onto the loops on the maxillary denture base. The loops on the denture were fabricated using 0.9 mm orthodontic wire (Figure 9).

**Figure 10:**
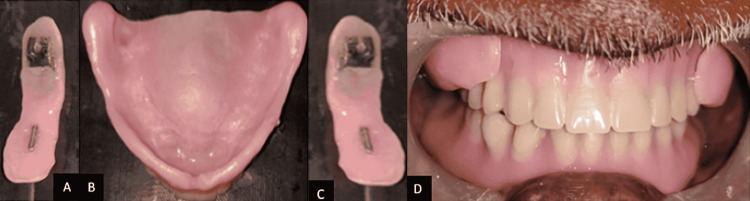
Finished and polished denture with detachable cheek plumper A: Right cheek plumper with attachment; B: Finished and polished complete dentures; C: Left cheek plumper with attachment; D: Finished and polished denture with detachable cheek plumper.

Step 5: Denture insertion - On the day of placement, the dentures and cheek plumper were tried in the patient's mouth (Figure 10).

**Figure 11:**
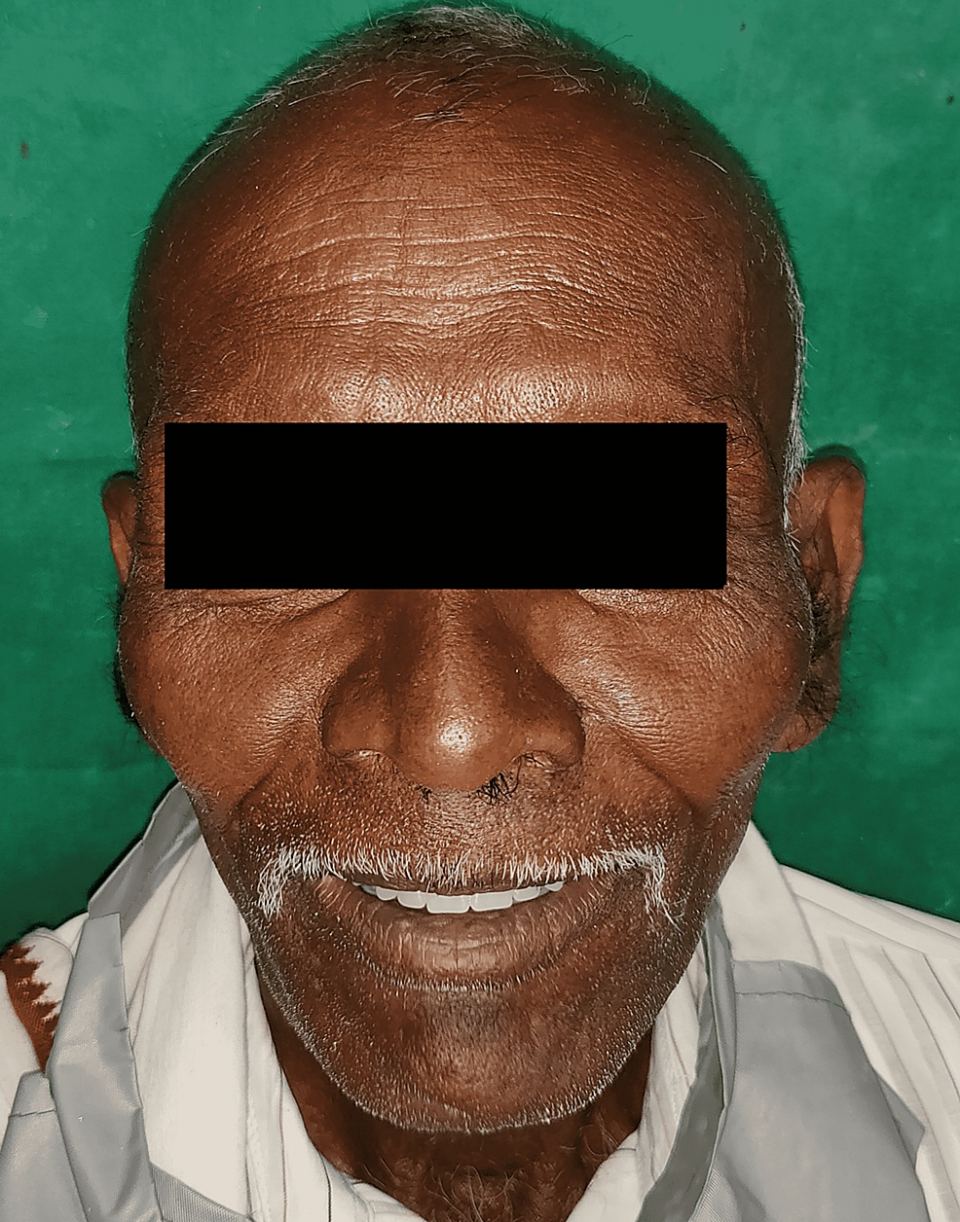
Post-op photograph

The retention, stability, and support of the maxillary and mandibular dentures were examined. The occlusion was confirmed, and the grinding was done selectively. The patient was comfortable with the complete denture prosthesis. The patient was taught how to use the dentures and cheek plumper. The patient was recalled after 24 hours and seven days, and the post-insertion problems were treated (Figure 11). Periodic recall visits were scheduled to verify the retention, comfort, and function.

## Discussion

Tooth loss will continue to be a concern in the near future, necessitating prosthetic replacement. To some extent, complete dentures restore aesthetics and function. While the majority of edentulous patients adjust well to their impairment and prosthesis, others have significant functional and psychological problems [4,11].

Special attention to specific problems like flabby tissue, severely resorbed ridges, and sunken cheeks needs to be given for each patient rather than the fabrication of a complete denture. There are surgical corrections available for these problems However, in most geriatric patients, due to chronic medical conditions or medical treatments, surgical procedures to excise the mobile mucosa, increase bone ridges, sinus lift, and utilise dental implants are insufficient. Also, the surgical procedure leaves a scar which is not acceptable; as a result, the negative aspects of the prosthetic field can be managed by expanding the basic principles of constructing complete dentures, rather than resorting to surgery [12]. The use of anatomic teeth in resorbed ridge cases is controversial, but there are several case reports in which patients successfully used dentures with anatomic teeth arranged in a balanced occlusion scheme to improve masticatory efficiency [5,13].

Similarly, various options for sunken cheeks are available, including modifying the denture. The incorporation of hooks and wire for mechanical retention of detachable cheek plumper also serves the purpose well. The major problem with the cheek plumper is the reduced denture stability and retention due to the increased weight of the maxillary denture. However, the use of detachable plumper reduces the weight, allows easy insertion and removal, and is also simple to clean. The patient can also choose to wear the denture without the cheek plumper.

## Conclusions

The case report describes the simple, novel technique for the fabrication of detachable cheek plumpers. Though cheek plumpers are commonly used, the technique described in the case report is novel and easy to fabricate. An effort was made to improve both the appearance and functionality by resolving the problem of the resorbed ridges and the flabby tissue. A complete denture with detachable cheek plumper attached to the orthodontic wire gave the patient a more youthful appearance with well-supported cheeks, which helped to increase the patient's self-confidence and provided outstanding stability during various functions. This novel strategy contributed to the patient's general well-being.
